# Antidepressant-like Effects of Chinese Quince (*Chaenomeles sinensis*) Fruit Based on In Vivo and Molecular Docking Studies

**DOI:** 10.3390/ijms25115838

**Published:** 2024-05-27

**Authors:** Dong Wook Lim, Guijae Yoo, Yun Tai Kim, Changho Lee

**Affiliations:** Division of Functional Food Research, Korea Food Research Institute, Wanju 55365, Republic of Korea; dwlim@kfri.re.kr (D.W.L.); gjyoo@kfri.re.kr (G.Y.); ytkim@kfri.re.kr (Y.T.K.)

**Keywords:** *Chaenomeles sinensis* fruit, polyphenol, monoaine oxidase B inhibitor, molecular docking

## Abstract

In this study, we examined the potential antidepressant-like effects of Chinese quince fruit extract (*Chaenomeles sinensis* fruit extract, CSFE) in an in vivo model induced by repeated injection of corticosterone (CORT)-induced depression. HPLC analysis determined that chlorogenic acid (CGA), neo-chlorogenic acid (neo-CGA), and rutin (RT) compounds were major constituents in CSFE. Male ICR mice (5 weeks old) were orally administered various doses (30, 100, and 300 mg/kg) of CSFE and selegiline (10 mg/kg), a monoamine oxidase B (MAO-B) inhibitor, as a positive control following daily intraperitoneal injections of CORT (40 mg/kg) for 21 days. In our results, mice treated with CSFE exhibited significant improvements in depressive-like behaviors induced by CORT. This was evidenced by reduced immobility times in the tail suspension test and forced swim test, as well as increased step-through latency times in the passive avoidance test. Indeed, mice treated with CSFE also exhibited a significant decrease in anxiety-like behaviors as measured by the elevated plus maze test. Moreover, molecular docking analysis indicated that CGA and neo-CGA from CSFE had stronger binding to the active site of MAO-B. Our results indicate that CSFE has potential antidepressant effects in a mouse model of repeated injections of CORT-induced depression.

## 1. Introduction

Hyperactivity of the hypothalamic–pituitary–adrenal (HPA) axis causes a continuous induction of stress hormones such as corticosterone (CORT) [1]. Excessive CORT activity increases monoamine oxidase (MAO) in astrocytes, leading to the degeneration of monoamine neurotransmitters such as serotonin or dopamine in the brain [2]. Indeed, MAO metabolism also generates reactive oxygen species (ROS) like hydrogen peroxide, resulting in neurotoxicity [3]. MAO inhibitors, selegiline, or safinamide are proposed as therapeutic agents for treating neurodegenerative disorders, such as depression, Parkinson’s, and Alzheimer’s diseases [4]. Despite the potent antidepressant and anti-neurodegenerative efficacy of MAO inhibitors, their clinical use has been limited due to their significant adverse effects, notably the hypertensive crisis known as the cheese effect [5]. Alternative methods of treating depression, such as using herbal remedies like *Hypericum perforatum*, commonly known as St. John’s wort [6], or consuming a diet rich in antioxidants from fruits and vegetables, have attracted attention as potential treatment options [7]. Additionally, according to the guidelines of the American Psychiatric Association (APA), consuming St. John’s wort extract has been reported to have a similar effect to antidepressants in treating patients with depression [8]. Therefore, there is a need for research on new antidepressants derived from various natural products, aiming for high efficacy and minimal side effects.

*Chaenomeles sinensis* Koehne also known as Chinese Quince is the fruit of a deciduous tree that belongs to the Rosaceae family. It is widely distributed in East Asia, including China, Japan, and Korea [9]. *C. sinensis* fruit is hard to eat due to a strong acidity and astringency, but is often processed and used in the preparation of fruit liquor, tea, jam, and candy [10]. Additionally, it has been used in traditional medicine to treat sore throat, diarrhea, and inflammation [11]. Recently, a randomized clinical trial demonstrated that quince syrup significantly improved the condition of patients with gastroesophageal reflux disease, whether in children or during pregnancy [12,13]. Several previous preclinical studies have shown that *C. sinensis* fruits exhibit anti-obesity [11], anti-inflammatory [14], antioxidant [15], anti-ulcerative colitis [16], anti-osteoarthritis [17], anti-hyperuricemic [18], anti-diabetic, including antihyperglycemic and antihyperlipidemic [19], and anti-aging [20] activities. These beneficial activities are mainly attributed to polyphenols, including phenolic acids, coumarins, and lignans from *C. sinensis* fruits [21]. Since the close relationship between oxidative stress and the pathophysiology of depression [22], several previous studies have indicated that consuming dietary polyphenols serves as a neuroprotective alternative to alleviate anxiety symptoms and manage mental disorders, including depression and anxiety [23]. However, there have been no detailed reports yet, at least as far as we know, on its efficacy in helping mental health, such as antidepressant-like effects of *C. sinensis* fruit extract (CSFE).

This study utilized a variety of behavioral tests, including the open field test and rotarod test, which are related to locomotor activity; the elevated plus maze, which is related to anxiety; the passive avoidance test, which is related to memory impairment; and the tail suspension test and forced swim test, which are related to depression, on corticosterone-induced depressive mice. Additionally, the active polyphenol compounds from CSFE were screened for their binding affinities in the active site of MAO-B using molecular docking analysis [24].

## 2. Results

### 2.1. Effect of CSFE on OFT

**Figure 1:**
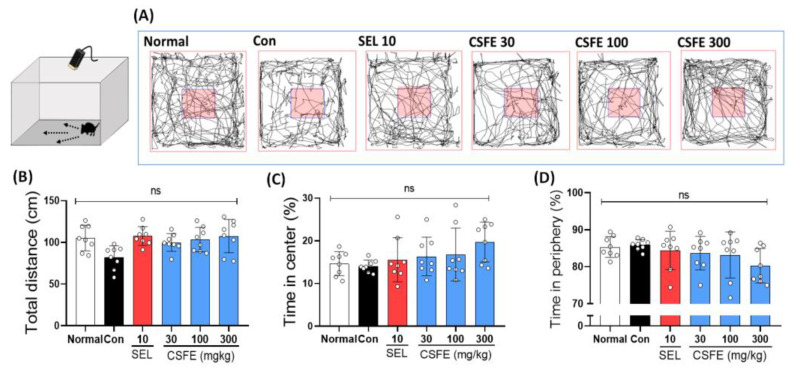
Investigation into the impact of CSFE on CORT-induced depressive mice during the OFT. A graphical representation of locomotor activity was recorded during a 5 min observation period in the OFT (**A**). The total distance traveled in the OFT (**B**) and the number of line crossings in both the center (**C**) and periphery (**D**) of the field were measured. No significant differences were noted among the groups in terms of locomotor activity. The results are presented as mean ± SD (*n* = 8, per group), and differences among experimental groups were assessed using analysis of variance (ANOVA). ns, non-significant, Con, control; CORT, corticosterone; SEL, selegiline; CSFE, *Chaenomeles sinensis* fruit extracts.

In our study, we investigated the effect of CSFE on locomotor activity in CORT-induced depressive mice. According to our tracing results, the CORT-injected groups did not exhibit a significant difference compared to the normal group in terms of total distance (cm), as well as time spent (%) in the center and periphery (Figure 1A). These findings are similar to previous reports indicating that chronic CORT injections in control male or female mice did not result in alterations in locomotor activity [25,26]. Our results also observed that there was no significant difference in locomotor activity among the groups, including the SEL- or CSFE-treated groups (Figure 1B–D).

### 2.2. Effect of CSFE on Rotarod

**Figure 2:**
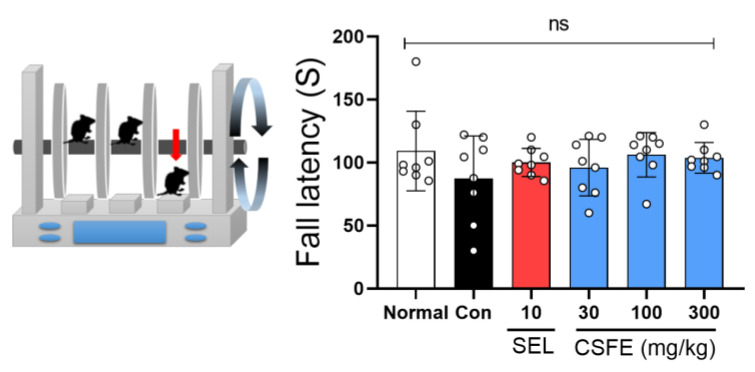
Effect of CSFE on the Rotarod in CORT-induced depressive mice. No significant behavioral alternations were observed among treatment groups on motor coordination in mice. Results are presented as mean ± SD (*n* = 8, per group). Differences among experimental groups were determined by analysis of variance (ANOVA) test. ns; non-significant, Con, control; CORT, corticosterone; SEL, selegiline; CSFE, *Chaenomeles sinensis* fruit extracts.

The Rotarod test was conducted to assess the impact of CSFE on motor coordination in mice induced with depressive symptoms by CORT. As shown in Figure 2, the CORT-injected control mice demonstrated no effect on fall latency (s) compared to the sham mice. Similarly, the groups treated with SEL or CSFE also exhibited no significant effect on motor coordination (Figure 2).

### 2.3. Effect of CSFE on EPM

**Figure 3:**
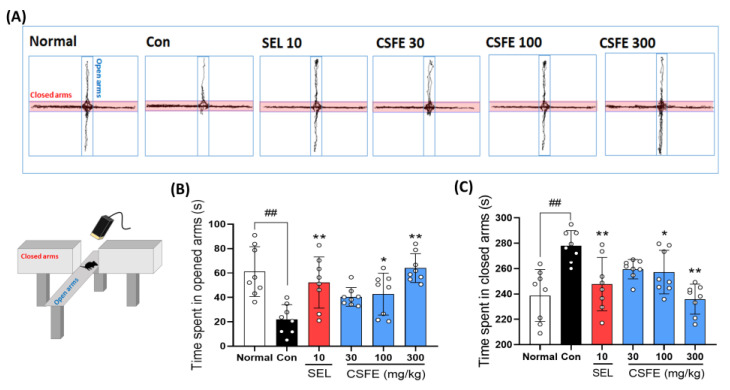
Evaluation of the impact of CSFE on the EPM in CORT-induced depressive mice. A graphical representation of locomotor activity was recorded during a 5 min observation period in the EPM (**A**). Treatment with CSFE resulted in a notable amelioration of CORT-induced anxiety-like behavior, characterized by a significant increase in the time spent in the open arms (**B**) and a decrease in time spent in the closed arms (**C**). Data are presented as mean ± SD (*n* = 8 per group), and differences among experimental groups were assessed using analysis of variance (ANOVA). ## *p* < 0.01 versus the Con group; * *p* < 0.05, and ** *p* < 0.01, versus the normal group. Con, control; CORT, corticosterone; SEL, selegiline; CSFE, *Chaenomeles sinensis* fruit extracts.

To explore the impact of CSFE on anxiety-like behavior induced by CORT, a key symptom of depression, we employed the EPM test, a well-established method for assessing anxiety-like behavior in mice. In the stress-induced anxiety model, mice displayed a preference for closed-arm movements and a reduced frequency of open-arm movements compared to sham mice [27]. As expected, our results showed that control mice injected with CORT spent significantly less time in the open arm and more time in the closed arm compared to the sham group (Figure 3A). However, treatment with CSFE led to a notable improvement in CORT-induced anxiety-like behavior, particularly at a dosage of 300 mg/kg (Figure 3B,C).

### 2.4. Effect of CSFE on PAT

**Figure 4:**
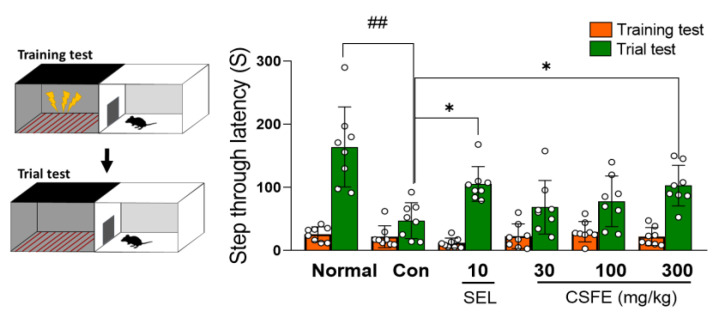
Impact of CSFE on CORT-induced depressive mice in the PAT. CORT-injected control mice displayed a significantly decreased step-through latency time (s), whereas treatment with CSFE at doses of 300 mg/kg resulted in a significant increase in latency time. Data are presented as mean ± SD (*n* = 8, per group), and differences among experimental groups were assessed using analysis of variance (ANOVA). ## *p* < 0.01 versus the normal group; * *p* < 0.05 versus the normal group. Con, control; CORT, corticosterone; SEL, selegiline; CSFE, *Chaenomeles sinensis* fruit extracts.

To investigate the effect of CSFE on CORT-induced cognitive dysfunction, specifically one of the depressive symptoms, we conducted a PAT. Previous research has demonstrated that treatment with SEL in mice restored the CORT-induced reduction in step-through latency time, which is consistent with our own findings [28]. Furthermore, our investigation demonstrates that treatment with CSFE significantly ameliorated the memory impairment induced by CORT, indicating its potential in mitigating depressive-like symptoms, such as memory deficits, in CORT-treated mice (Figure 4).

### 2.5. Effect of CSFE on TST and FST

**Figure 5:**
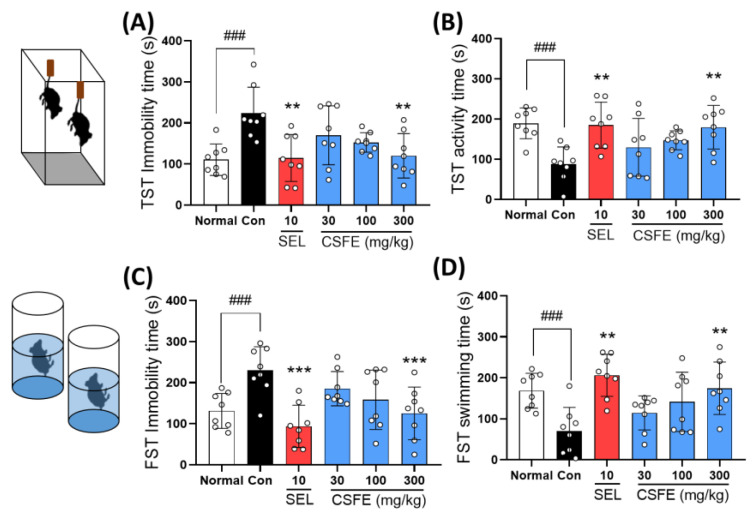
Effect of CSFE on the TST and FST in CORT-induced depressive mice. Mice treated with CSFE at doses of 300 mg/kg exhibited significant decreased immobility (**A**,**C**) times (s) and increased activity (**B**,**D**) times (s). Results are presented as mean ± SD (*n* = 8, per group). Differences among experimental groups were determined via analysis of variance (ANOVA) test. ### *p* < 0.001 versus the normal group; ** *p* < 0.01, and *** *p* < 0.001 versus the Con group. Con, control; CORT, corticosterone; SEL, selegiline; CSFE, *Chaenomeles sinensis* fruit extracts.

The TST and FST are behavioral experiments primarily employed to assess depressive behavior, where an increase in immobility time under isolated conditions is considered indicative of depression-like behavior. In our results, control mice subjected to repeated CORT injections exhibited a notable increase in immobility time compared to the normal control group. However, mice treated with SEL or CSFE showed a significant decrease in immobility time compared to the control group (Figure 5). These findings suggest that CSFE demonstrated efficacy in alleviating depression in CORT-induced depressive mice.

### 2.6. Molecular Docking Analysis

**Figure 6:**
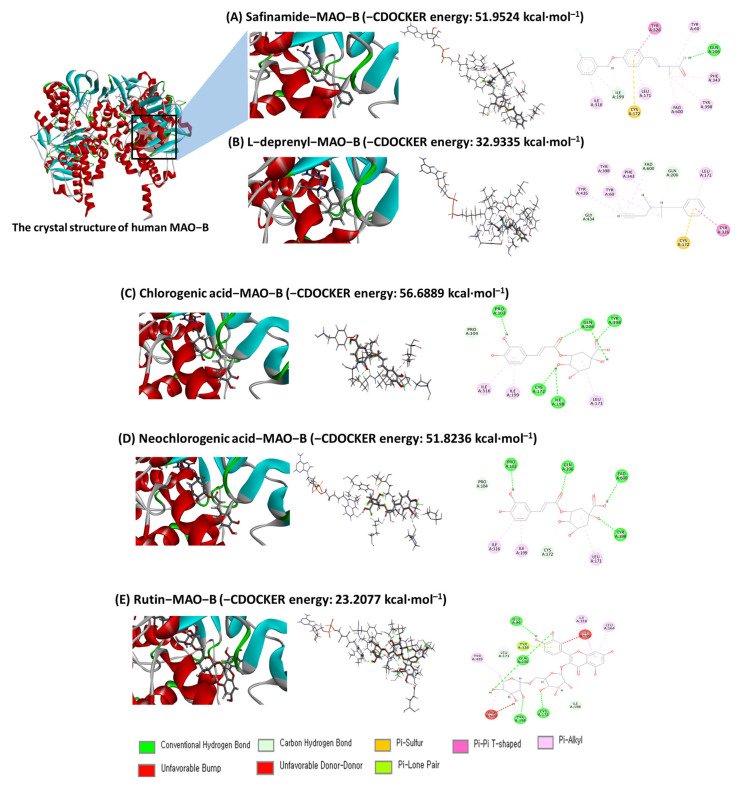
Molecular docking analysis of the interaction of the ligands at the active site of MAO-B (PDB code: 2V5Z). Safinamide—MAO-B (**A**), L-deprenyl—MAO-B (**B**), Chlorogenic acid—MAO-B (**C**), Neochlorogenic acid—MAO-B (**D**), and Rutin—MAO-B (**E**).

Molecular docking analysis was performed to investigate the interaction between MAO-B and phenolic compounds, CGA, neo-GCA, and RT from CSFE. It was found that the phenolic compounds from CSFE fit well at the active site of MAO-B as in the case of native co-crystallized ligand inhibitor safinamide. The docking method was validated initially via re-docking of safinamide at the active site of MAO-B. As control compounds, the co-crystallized ligand safinamide and the selective MAO-B inhibitor L-deprenyl were used. The binding affinity of CA and NCA were −56.6889 and −51.8236 kcal/mol for MAO-B, respectively, (Figure 6C,D) and are comparable to the reference inhibitor, safinamide which showed a binding affinity of −51.9524 kcal/mol (Figure 6A). Additionally, RT also showed similar binding affinity to the MAO-B inhibitor L-deprenyl (−23.2077 and −32.9335 kcal/mol, respectively) (Figure 6B,E). The key residues involved in MAO-B inhibition were found to be LEU-171, ILE-199, TYR-326, and GLN-206, which most contribute to the stability of the inhibitor when binding to MAO-B [29]. CGA showed interaction with amino acid residues PRO102, PRO104, LEU171, CYS172, ILE198, ILE199, GLN206, ILE316, and TYR398. Similarly, neo-CGA showed interaction with PRO102, PRO104, LEU171, CYS172, ILE199, GLN206, ILE316, TYR398, and FAD600. RT showed interaction with amino acid residues GLU84, LEU164, LEU171, CYS172, ILE198, ILE199, GLN206, ILE316, TYR326, TYR398, TYR435, FAD600 at the active site of MAO-B (Table 1). These results suggest that standard compounds from CSFE may be on par or better than previously known inhibitors, safinamide or L-deprenyl, regarding MAO-B inhibition.

## 3. Discussion

While the pathogenesis of depression is not yet fully understood, HPA axis dysfunction has been identified as an important risk factor [30]. HPA axis dysfunction results in dysregulation of the negative feedback on glucocorticoid levels, such as cortisol. This leads to excessive cortisol levels, as observed in numerous patients with depression [31]. Elevated cortisol levels stimulate MAO activity in astrocytes within the central nervous system [32]. MAO-induced ROS generation within the nervous system during this process inhibits the production of neurotransmitters, such as serotonin and dopamine [33]. The depression-like behavior induced by CORT in mice faithfully recapitulates HPA-axis dysfunction [34]. Repeated injection of CORT at a dose of 40 mg/kg resulted in depressive-like behavior, characterized by increased immobility time in the FST and TST, mimicking disease phenotypes in humans. However, no difference in locomotor activity was noted [35]. These behaviors can be significantly improved by treatment with antidepressant drugs or natural products [36]. Similarly, our results showed that mice injected daily with 40 mg/kg CORT for three weeks exhibited significantly increased immobility times in the TST and FST, indicative of depressive behavior when compared to sham mice. However, the CSFE-treated group showed significantly improved immobility and activity times in the TST and FST without any changes in locomotor activity. These findings indicate that CSFE possesses antidepressant properties in animal models of CORT-induced depression.

Memory impairment is a highly prevalent and severe symptom in patients with depression [37]. Mice with CORT-induced depression show a significantly reduced step-through latency time compared to normal mice in the PAT test [38]. This behavior indicates impairment of neuronal function and associated memory deficits, which can be significantly improved by the administration of antidepressant drugs or natural products [39]. As expected from our results, mice in the CORT-treated control group showed memory impairment, reflected by a significant decrease in step-by-step latency time, whereas the group treated with high doses of CSFE showed a significant increase in PAT latency. These results demonstrated that CSFE can enhance memory function in mice with CORT-induced depression.

Anxiety disorders are closely related to depression [40], and this relationship has been well established in studies of both animal models and patients [41]. Antidepressant drugs, such as fluoxetine, can alleviate anxiety [42]. The EPM is a widely used behavioral analysis method for evaluating the efficacy of anti-anxiety treatments in rodents and for elucidating the mechanisms underlying anxiety-related behaviors [43]. EPM equipment generally consists of two arms divided into open and closed sections. Increased anxiety behavior is typically associated with significantly reduced time spent in the open arms [44]. In our EPM results, the control CORT-treated mice exhibited significantly increased anxiety-like behavior, spending significantly less time in the open arms and more time in the closed arms. However, the CSFE-treated group showed significant improvement in CORT-induced anxiety-like behaviors. These results suggest that CSFE has anxiolytic effects in mice with CORT-induced anxiety.

Our HPLC analysis revealed CGA, neo-CGA, and RT as the major components of CSFE. Natural products, including phytochemicals, are known to possess high antioxidant capacities and demonstrate beneficial effects in various oxidative stress-related neuropsychiatric disorders, such as depression [45,46]. Preclinical research has shown that CGA, the main active compound found in *Eriobotrya japonica* and *Crataegus pinnatifida* fruits, improves CORT-induced depressive behavior by preventing MAO-B activation following ROS production [47,48]. Dicaffeoylquinic acid in Arctium lappa alleviates CORT-induced depressive behaviors, including memory loss, by inhibiting MAO-A and MAO-B activity in neurons and astrocytes [28]. Neo-CGA from Actinidia argute and kiwifruit was shown to exert neuroprotective effects in PC-12 and SH-SY5Y cell lines through the inhibition of acetylcholinesterase and butyrylcholinesterase [49]. RT exhibited antidepressant activity in rats with reserpine-induced anxiety and depression by reducing acetylcholinesterase levels [50]. It is therefore possible that CA, neo-GCA, and RT are the active compounds that play major roles in the antidepressant-like effects of CSFE. However, further research should be conducted to determine the efficacy of CSFE, the role of its active components in relieving depression, as well as its exact mechanism of action.

Molecular docking is a useful computational method to predict the binding of small molecules to proteins. It facilitates the identification of potential binding modes and the understanding of the mechanisms involved [51]. Molecular docking analysis has been used to develop potent MAO-B inhibitors from natural products [52]. Our molecular docking studies revealed that, among the major components identified via HPLC analysis, CGA and neo-CGA were predicted to interact with MAO-B by strongly binding to its active site, to an extent comparable to the reference inhibitor safinamide. Thus, we hypothesized that CGA and neo-CGA are the main active components of CSFE, leading to MAO-B inhibition.

In summary, our results indicated that CSFE exerts antidepressant-like effects in CORT-treated mice. These are characterized by reduced immobility times without an effect on locomotor activity, in addition to significantly alleviated memory impairment.

## 4. Materials and Methods

### 4.1. Sample Preparation and HPLC Analysis

**Figure 7:**
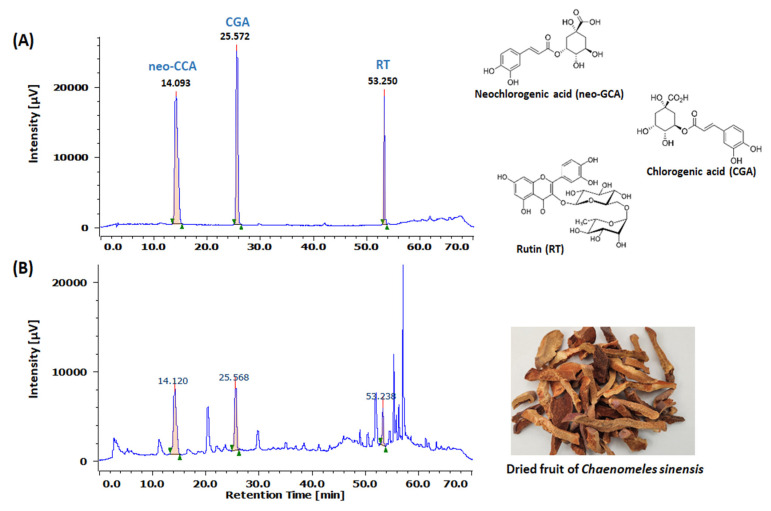
HPLC chromatogram of neochlorogenic acid (neo-CGA), chlorogenic acid (CGA), and rutin (RT) as a standard compound’s mixture (**A**) and the dried fruit of *C. sinensis* extract (CSFE) (**B**). The concentration of neo-GCA, CGA, and RT were 0.52 ± 0.005, 0.38 ± 0.006, and 0.53 ± 0.015 mg/g CSFE, respectively.

The dried fruit of *Chaenomeles sinensis* was obtained from the Jecheon herbal medicine market from South Korea. A total of 600 g of dried fruits of *C. sinensis* were soaked in 6000 L of 50% MeOH and extracted using a reflux device at 50 °C for 6 h. Following solvent removal via rotary evaporation, the resulting extract was freeze-dried (CSFE), yielding 12.1% (*w*/*w*). CSFE was dissolved in HPLC-grade MeOH, filtered using a 0.45 µm PVDF Syringe Filter, and prepared for analysis. Samples were analyzed using an HPLC system (Jasco, Tokyo, Japan) equipped with an YMC Hydrosphere C18 column (4.6 mm × 250 mm, 3.0 μm, YMC, Tokyo, Japan). The mobile solvent phase consisted of 0.2% formic acid (A) and MeOH (B), and the proportion of solvent (A) was first reduced from 85% to 20% within 60 min and then increased to 85%. The total analysis time is 70 min and the injection volume of the analysis sample was 10 μL. The flow rate was set at 1 mL/min, and sample detection was performed at 305 nm. Neochlorogenic acid (neo-CGA), chlorogenic acid (CGA), and rutin (RT) were purchased from Sigma Aldrich (St. Louis, MO, USA) as standard components for analysis and dissolved in MeOH to prepare stock solutions (10 mg/mL) stored at 4 °C. As a result of quantitative analysis repeated three times, it was revealed that the concentrations of neo-CGA, CGA, and RT were 0.52 ± 0.005, 0.38 ± 0.006, and 0.53 ± 0.015 (mean ± SD) mg/g, respectively. Figure 7 shows a representative chromatogram of the reference compounds with their corresponding peaks in the CSFE.

### 4.2. Experimental Animals and Sample Treatment

**Figure 8:**
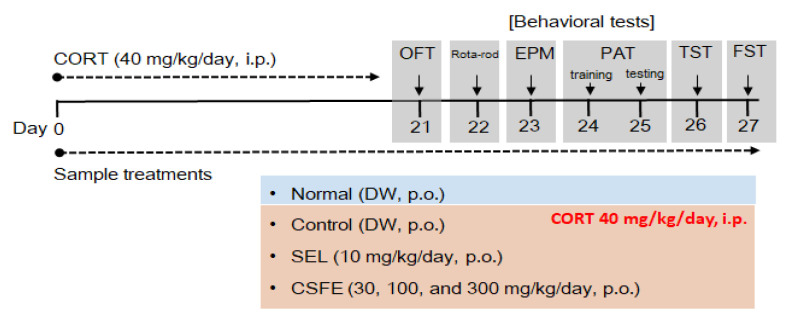
Experimental design and behavioral experiment. CORT, corticosterone; DW, distilled water; OFT, open-field test; EPM, elevated plus maze; PAT, passive avoidance test; TST, tail suspension test; FST, forced swim test; SEL, selegiline; CSFE, *C. sinensis* fruit extract.

Male ICR mice (5 weeks old) were obtained from KOATECH Animal Inc. (Pyeongtaek, Republic of Korea). All mice underwent an adaptation period of at least 1 week before behavioral experiments and were housed in accordance with the guidelines of the Korea Food Research Institute Animal Care and Use Committee (IACUC, KFRI-M-19016). The housing environment maintained a temperature control of 21 ± 2 °C and a light/dark cycle set to 12 h. To induce depression-like behavior in mice, repeated intraperitoneal (i.p.) injections of corticosterone (CORT, Sigma Aldrich, St. Louis, MO, USA) at a dose of 40 mg/kg were administered for 3 weeks according to the protocol of a previous report [28,53]. The mice were randomly divided into six groups with the following compositions: (1) normal (non-CORT i.p. + vehicle p.o.), (2) control (CORT i.p. + vehicle p.o.), (3) SEL (CORT i.p. + selegiline 10 mg/kg p.o.), (4) CSFE 30 (CORT i.p. + CSFE 30 mg/kg p.o.), (5) CSFE 100 (CORT i.p. + CSFE 100 mg/kg p.o.), and (6) CSFE 300 (CORT i.p. + CSFE 300 mg/kg p.o.). CORT was dissolved in a 0.9% saline solution containing 1% dimethyl sulfoxide and 1% Tween 80. SEL and CSFE were dissolved in distilled water and used according to the prescribed dosage. All samples were orally administered to mice once a day for 3 weeks, and each behavioral experiment was conducted according to the schedule outlined in Figure 8. Behavioral experiments were performed 1 h after oral administration of the samples to the mice.

### 4.3. Open Field Test (OFT)

The OFT is a behavioral experiment mainly used to analyze the general motor activity and exploration of rodents [54]. Specifically, it is employed to observe how anti-anxiety and antidepressant agents influence locomotor activity [55]. The mouse was placed in the central area of the open maze (50 × 50 × 50 cm), and its free movement was recorded using a video camera for 5 min. The locomotor activity was analyzed using the SMART video tracking system (SMART v3.0, Panlab SL, Barcelona, Spain), measuring the total distance the mouse moved (in centimeters) and the spend time (%) in the central and peripheral areas.

### 4.4. Rotarod Test

The rotarod test is widely used to assess motor coordination in rodents. It was performed following procedures established in previous studies. Briefly, the mouse was placed on a rod with a diameter of 3 cm on a rotarod (Ugo Basile, Varese, Italy), and the rotation was gradually increased from 2 to 20 rpm over 300 s. The latency until the mouse fell was recorded.

### 4.5. Elevated Plus Test (EPT)

The elevated plus maze test is one of the most widely used behavioral tests to assess anxiety-like behavior in rodents [56]. The apparatus for the elevated plus maze test is 50 cm high and has a cross shape, consisting of two closed arms and two open arms. Initially, the mouse is placed in the central area at the intersection of the elevated plus maze and allowed to freely explore the maze for a set period of 10 min. When the mice freely moved within the EPM equipment for 10 min, the durations they spent in the opened arms and the closed arms were measured to compare anxiety-related behavioral patterns. The total movements of the mouse were recorded with a video camera and analyzed using the SMART video tracking system (SMART v3.0, Panlab SL, Barcelona, Spain).

### 4.6. Passive Avoidance Test (PAT)

The passive avoidance task (GEMINI avoidance system, SD Apparatus, San Diego, CA, USA) comprises a bright chamber with a centrally positioned light and an automatically opening guillotine-shaped door, alongside a dark chamber featuring continuous electrical stimulation (Size: 24 × 20 × 20 cm). Initially, during the training phase, the mouse was placed into the brightly lit chamber. After a 1 min adaptation period, the guillotine door opened automatically, allowing the mouse to enter the dark chamber. Subsequently, the door closed automatically, and the mouse received a 0.5 mA electric shock for 3 s. The next day, the trial phase was conducted similarly to the training phase, except without the electric shock. The cognitive-related behaviors, measured by the step-through latency time, presenting the time taken for the mouse to enter the dark chamber from the bright chamber, were recorded for up to 300 s. If the mouse failed to enter the dark chamber within 300 s, it was excluded from the data analysis.

### 4.7. Tail Suspension Test (TST)

The TST is a commonly used method to evaluate the efficacy of potential antidepressant agents in animal behavior research [57]. We utilized equipment equipped with an auto sensor attached to the TST apparatus (BioSeb, Chaville, France) to measure the movement of the mouse automatically. Adhesive tape was applied to each mouse’s tail, which was then connected to a hook with a sensor. The total immobility time was recorded over a period of 6 min.

### 4.8. Forced Swim Test (FST)

Widely recognized as a key method for investigating depressive-like behavior in rodents, the FST serves as a pivotal tool in assessing the antidepressant-like effects of CSFE and its constituents, following a standardized protocol. Mice were immersed in a transparent cylinder filled with water at a consistent depth (10 cm) and temperature range (22–24 °C) for a duration of 6 min. Analysis of immobility and swimming behavior during the final 4 min was conducted using SMART version 3.0 software (Panlab SL, Barcelona, Spain), consistent with established procedures.

### 4.9. Molecular Docking Study

Molecular docking study was performed using CDOCKER module in Discovery Studio (DS) 23.1 Software (BIOVIA Co., Ltd., San Diego, CA, USA). CDOCKER is a molecular dynamics based on the Chemistry at Harvard Macromolecular Mechanics (CHARMm) algorithm [58]. The high-resolution crystal structure of human monoamine oxidase B (MAO-B) in complex with safinamide (PDB code: 2V5Z; Resolution 1.6 Å) was retrieved from the Protein Data Bank (PDB, http://www.rcsb.org/pdb/home/home.do, accessed on 7 March 2024). Native co-crystallized ligand safinamide was used to define the active site of the target protein and the MAO-B enzyme was prepared by the “Prepare Protein” tool in DS software. Incomplete amino acid residues, undetected side chains and loops were supplemented and the hydrogens were added in the protein, energy minimization was performed using the CHARMm force field. Native co-crystallized ligand safinamide, the selective MAO-B inhibitor L-deprenyl and neochlorogenic acid, chlorogenic acid, and rutin isolated from *C. sinensis* were docked in a flexible manner to achieve a more realistic view of the possible protein–ligand interactions. The result was given by –CDOCKER energy, a score calculated including the protein–ligand interaction energy and the ligand strain energy. The score of the substrate ligand is one of the criteria for choosing a docked pose, and higher values indicate more favorable binding [59].

### 4.10. Statistical Analysis

The data were presented as mean ± standard deviation and analyzed using one-way analysis of variance (ANOVA), followed by Dunnett’s test for post-hoc comparisons, employing the Prism 8 software (GraphPad Software v8.0, Inc., San Diego, CA, USA). Significance was assessed at a threshold of *p* < 0.05.

## 5. Conclusions

The results of our study suggest that the CSFE formulation, which is mainly composed of CGA, neo-CGA, and RT, effectively alleviates stress hormone-induced depressive and anxiety-like behaviors, as confirmed by a series of behavioral tests. Further, the main active compounds of CSFE, namely CGA and neo-CGA, exhibited strong binding to the active site of MAO-B. Taken together, CSFE may represent a promising candidate for the treatment of depression. However, further studies are needed to clearly understand the mechanism by which the three major phenolics we presented, CGA, neo-CGA, and RT, exhibit antidepressant-like effects in CSFE.

## Figures and Tables

**Table 1:** Docking score and interaction of five ligands with MAO-B.

Ligands	-CDOCKER Energy (kcal·mol^−1^)	Amino Acid Residues Interacting with Ligands
Safinamide	51.9524	TYR60, LEU171, CYS172, ILE199, GLN206, ILE316, TYR326, PHE343, TYR398, FAD600
L-deprenyl	32.9335	TYR60, LEU171, CYS172, GLN206, TYR326, PHE343, TYR398, GLY434, TYR435, FAD600
Chlorogenic acid	56.6889	PRO102, PRO104, LEU171, CYS172, ILE198, ILE199, GLN206, ILE316, TYR398
Neochlorogenic acid	51.8236	PRO102, PRO104, LEU171, CYS172, ILE199, GLN206, ILE316, TYR398, FAD600
Rutin	23.2077	GLU84, LEU164, LEU171, CYS172, ILE198, ILE199, GLN206, ILE316, TYR326, TYR398, TYR435, FAD600

## Data Availability

The data presented in this study are available on request from the corresponding authors.
